# Lower Protein-to-Carbohydrate Ratio in Maternal Diet is Associated with Higher Childhood Systolic Blood Pressure up to Age Four Years

**DOI:** 10.3390/nu7053078

**Published:** 2015-04-24

**Authors:** Michelle L. Blumfield, Caryl Nowson, Alexis J. Hure, Roger Smith, Stephen J. Simpson, David Raubenheimer, Lesley MacDonald-Wicks, Clare E. Collins

**Affiliations:** 1 School of Health Sciences, Faculty of Health and Medicine, University of Newcastle, Callaghan, New South Wales 2308, Australia; E-Mails: Michelle.Blumfield@newcastle.edu.au (M.B.); Lesley.Wicks@newcastle.edu.au (L.M.-W.); 2 Priority Research Centre in Physical Activity and Nutrition, Faculty of Health and Medicine, University of Newcastle, Callaghan, New South Wales 2308, Australia; 3 School of Exercise and Nutrition Science, Deakin University, Geelong, Victoria 3216, Australia; E-Mail: Caryl.Nowson@deakin.edu.au; 4 School of Medicine and Public Health, Faculty of Health and Medicine, University of Newcastle, Callaghan, New South Wales 2308, Australia; E-Mail: Alexis.Hure@newcastle.edu.au; 5 Mothers and Babies Research Centre, Hunter Medical Research Institute, John Hunter Hospital, Level 3, Endocrinology, Locked Bag 1, Hunter Region Mail Centre, New South Wales 2310, Australia; E-Mail: Roger.Smith@newcastle.edu.au; 6 Charles Perkins Centre, School of Biological Sciences, University of Sydney, Sydney, New South Wales 2006, Australia; E-Mails: Stephen.Simpson@sydney.edu.au (S.J.S.); David.Raubenheimer@sydney.edu.au (D.R.)

**Keywords:** maternal, pregnancy, dietary intake, macronutrient, blood pressure, systolic, child, protein, diet, nutrition

## Abstract

The prenatal environment can influence development of offspring blood pressure (BP), which tracks into adulthood. This prospective longitudinal study investigated whether maternal pregnancy dietary intake is associated with the development of child BP up to age four years. Data are from 129 mother-child dyads enrolled in the Women and Their Children’s Health study. Maternal diet was assessed using a validated 74-item food frequency questionnaire at 18 to 24 weeks and 36 to 40 weeks, with a reference period of the previous three months. Child systolic and diastolic BP were measured at 3, 6, 9, 12, 24, 36 and 48 months, using an automated BP monitor. Using mixed-model regression analyses adjusted for childhood growth indices, pregnancy intakes of percentage of energy (E%) polyunsaturated fat (β coefficient 0.73; 95% CI 0.003, 1.45; *p* = 0.045), E% omega-6 fatty acids (β coefficient 0.89; 95% CI 0.09, 1.69; *p* = 0.03) and protein-to-carbohydrate (P:C) ratio (β coefficient −14.14; 95% CI −27.68, −0.60; *p* = 0.04) were associated with child systolic BP trajectory up to 4 years. Child systolic BP was greatest at low proportions of dietary protein (<16% of energy) and high carbohydrate (>40% of energy) intakes. There may be an ideal maternal macronutrient ratio associated with optimal infant BP. Maternal diet, which is potentially modifiable, may play an important role in influencing offspring risk of future hypertension.

## 1. Introduction

The worldwide burden of cardiovascular disease (CVD) has substantially increased, to cause an estimated 17.3 million deaths in 2008 [1]. It is forecast that by 2030, more than 23 million people will die annually from CVDs [2]. Elevated blood pressure (BP) and childhood adiposity are independent predictors of adult hypertension and CVD [3]. Both obesity and hypertension are increasingly prevalent in the pediatric population, with 42 million children under five estimated to be overweight in 2013 and 2% to 5% of children considered hypertensive globally [2]. There is strong evidence across diverse populations for tracking of BP from childhood into adulthood [4]. Even short periods of hypertension in childhood increases the adult risk of hypertension [1]. Thus early prevention of elevated BP is an important global public health goal.

Variations in maternal diet during pregnancy may lead to elevated offspring risk in adulthood for hypertension and obesity. Both human and animal studies provide compelling evidence suggesting a link between maternal nutritional status and offspring cardiovascular function [5]. Animal studies provide indisputable evidence that maternal protein restriction or a global reduction in dietary intake during pregnancy can influence offspring cardiovascular function [5,6,7,8]. Studies have mainly focused on interventions in pregnant rodents, although several dietary intervention studies in pregnant sheep suggest commonality among species [5]. Raised blood pressure has frequently been reported in offspring of nutritionally deprived animals and those fed a low protein-to-carbohydrate (P:C) ratio diet during pregnancy [5,9]. Prenatal exposure to protein restriction or dietary global restriction in animal models has produced offspring with abnormalities in vascular function in isolated resistance arteries [5]. However in recent cross-sectional studies of children born in developed countries, associations between changes in diet during pregnancy with childhood BP have been small, or negligible [10]. A recent Finnish study found systolic BP in four year old children was positively associated with maternal pregnancy carbohydrate and fat intakes [11]. The same group also reported a U-shaped relationship between both maternal carbohydrate and monounsaturated fat intakes and infant BP at six months [12].

The escalation in prevalence of maternal and child obesity increases the public health significance of determining the extent to which maternal diet can predict offspring BP [10]. In healthy weight pregnant women exposed to a variety of dietary and/or supplement regimes there was minimal impact on offspring BP, compared to women who experienced lifestyle and nutritional limitations similar to conditions in World War II [12,13]. However, much stronger associations have been reported on later BP when birthweight and/or accelerated postnatal growth were considered, or adjustment for current body weight [13]. This highlights the inter-relationships between childhood growth, current body weight and cardiovascular control.

Our previous work identified that across a wide range of species optimal ratios of macronutrients exist and that protein intake is a critical determinant of total energy intake [14,15]. Animal models confirm that the ratio of dietary P:C impacts on aging and disease, with indirect evidence from human models suggesting the P:C ratio is a primary determinant of health [16]. We have demonstrated a link between maternal dietary ratio of protein to non-protein energy to variations in offspring body composition [15]. In human pregnancy, maternal macronutrient profile was associated with fetal adiposity and fat distribution [14]. Here we have examined the link between maternal macronutrient ratios and childhood blood pressure trajectory. We hypothesized that after adjustment for potential confounders and growth indices, child systolic BP up to age four years would be associated with maternal pregnancy macronutrient intake and protein-to-carbohydrate (P:C) ratio.

## 2. Experimental Section

### 2.1. Sample

The sample is from the Women and Their Children’s Health (WATCH) study (*n* = 179). WATCH is a prospective, longitudinal cohort investigating whether maternal nutrition is an important predictor of offspring outcomes including growth, body composition and childhood cognition [17]. The WATCH study was initiated in July 2006 in Newcastle, Australia. Pregnant participants were recruited from the antenatal clinic at the John Hunter Hospital from July 2006 to June 2008. A consent rate of 61% was achieved for pregnant women who were approached [18]. No exclusion criteria were used during recruitment. Women attended study visits during pregnancy at 19, 24, 30, and 36 weeks of gestation. Postnatal follow-up of women and their children occurred at 3-month intervals during the first year after birth and annually thereafter, until age 4 years.

The characteristics of the WATCH cohort have been reported previously [19,20]. The WATCH study contained a higher proportion of women with post-school qualifications and socioeconomic advantage, but a similar proportion of overweight/obese and of indigenous ethnicity as the Australian population [14,20]. Significant differences were also previously found between women with available dietary data (*n* = 156) and women without dietary data (*n* = 23). Women without dietary data were less likely to be married or in a de facto relationship (*p* = 0.03) and more likely to be of socio-economic disadvantage (*p* = 0.04) and to have had a preterm delivery (*p* = 0.001) [14].

Dietary and BP data from 129 mother-child dyads were available for the current analysis. All participants gave written informed consent and ethical approval for the study was obtained from the Hunter New England Human Research Ethics Committee (06/05/24/5.06). Recruitment, withdrawals and participant attendance during pregnancy has previously been described [20].

### 2.2. Data Collection

Details of the data collection have been described elsewhere [20]. Demographic and social data were collected during the first study visit using questions modeled on those in the Women’s Health Australia, Australian Longitudinal Study on Women’s Health survey. Pre-pregnancy weight was self-reported at either the first antenatal clinic visit at 14 weeks gestation or at the first study visit. Dietary data during pregnancy were collected between 18 to 24 weeks and again at 36 to 40 weeks gestation using a validated 74-item food frequency questionnaire (FFQ), the Dietary Questionnaire for Epidemiological Studies. This tool was previously validated against weighed-food records in young women [21]. The FFQ includes food and beverage data but does not ask about vitamin or mineral supplement use. The dietary intake reference period was the previous three months. Therefore, dietary data collected between 18 to 24 weeks and 36 to 40 weeks gestation referred to a reference period of 6 to 24 weeks gestation (early pregnancy) and 24 to 40 weeks gestation (late pregnancy), respectively. Positive moderate to strong pairwise correlations have been previously reported between all dietary variables in early and late pregnancy (0.46 < *r* < 0.78; *p* < 0.001) [14]. Therefore, maternal dietary intakes during pregnancy were expressed as the mean of intakes during early and late pregnancy.

Child systolic and diastolic BP were measured at each postnatal study visit using an automated DINAMAP PRO 300V2 BP monitor (GE Healthcare, Helsinki, Finland) under standardised conditions. Maternal and child anthropometric measurements were taken at every study visit by a team of Accredited Practising Dietitians, each with Level One Anthropometry certification from the International Society for the Advancement of Kinanthropometry (ISAK). Weight and height were measured in accordance with the ISAK protocol.

### 2.3. Statistical Analysis

The main outcome measures were child systolic and diastolic BP (mm/Hg). Dietary predictors included maternal intake of energy yielding nutrients (energy, protein, total fat, saturated fatty acids (SFA), monounsaturated fatty acids, polyunsaturated fatty acids (PUFA), omega-3 (*n*-3) fatty acids, omega-6 (*n*-6) fatty acids, total carbohydrate, sugars, starch and fibre) expressed as total intakes (grams), percentage of total energy intake, and the ratio of P:C intake and *n*-6:*n*-3 intake. Nutrient intakes were adjusted for energy using the residual method [22]. Childhood growth indices included birthweight and BMI *z*-score. BMI was converted to *z*-scores using the WHO Child Growth Standards reference data [23].

Normally distributed data were reported as mean ± SD and non-parametric data as median, 25th and 75th percentiles. Comparisons were performed using two-sample *t*-tests, the Kruskal-Wallis test or the chi-squared statistic. Linear mixed-models used longitudinal data to determine whether maternal macronutrient intakes during pregnancy were associated with the development of childhood BP up to 4 years. Independent predictor variables were assessed for colinearity.

In preliminary univariate analyses, maternal age, pre-pregnancy weight, education, parity, smoking status, weight gained during pregnancy, preterm delivery, marital status, ethnicity and child gender were not significant predictors for childhood systolic or diastolic BP (*p* < 0.2) and did not remain in multivariate models (data not shown). Analyses were conducted using: (i) unadjusted data; (ii) data adjusted for maternal energy intake and child birthweight; and (iii) data adjusted for maternal energy intake, birthweight and child BMI *z*-score. All analyses were repeated with and without women who reported diabetes or hypertension during pregnancy. The main findings did not change with the exclusion of women who reported diabetes (*n* = 8) or hypertension (*n* = 10) during pregnancy, thus analyses were not adjusted for these conditions. To address potential dietary misreporting, the pregnancy energy cut-off values recommended by Meltzer *et al.* (2008) [24] were applied, by excluding those who reported daily energy intakes <4.5 or >20.0 MJ/day [24].

Parametric response surfaces for mean systolic BP were fitted over macronutrient intake arrays and then visualized by using nonparametric thin-plate splines. This approach allowed the complex relationship between infant systolic BP up to four years and the two major axes of percentage protein and percentage carbohydrate in the maternal diet to be visualized.

All data manipulation and statistical analyses were performed using Intercooled Stata 11.2 (Stata, College Station, Texas, USA). Graphics were performed using *R* software. *p*-Values < 0.05 were considered statistically significant.

## 3. Results

The final sample included 129 mother and child dyads. Maternal characteristics are presented in Table 1. There were no significant differences between individuals in this sub-study (*n* = 129) and the total sample of women and children in the WATCH cohort (*n* = 179).

**Table 1:** Maternal characteristics of the participants in the Women and Their Children’s Health Study.

Characteristic	Women (*n* = 129)
Age (year)	29.1 ± 5.4 ^1^
Height (cm)	165.1 ± 6.5
Born in Australia [*n* (%)] ^2^	120 (93.0)
Aboriginal, not Torres Strait Islander [*n* (%)]	4 (3.1)
Married or in de facto relationship [*n* (%)]	114 (88.4)
Education [*n* (%)] ^3^	96 (74.4)
Socioeconomic status, IRSAD ^4^ decile <= 5 [*n* (%)] ^5^	37 (28.7)
Smoked during pregnancy [*n* (%)]	15 (11.6)
Prepregnancy weight (kg)	65.0 (58.0, 79.0) ^6^
Weight gain during pregnancy (kg)	12.0 (8.9, 17.0)
Nulliparous [*n* (%)]	59 (45.7)
Preterm delivery before 37 weeks of gestation [*n* (%)]	9 (7.0)

^1^ Mean ± SD (all such values); ^2^ Other countries include England (*n* = 3), Belgium (*n* = 1), Canada (*n* = 1), Malaysia (*n* = 1), New Zealand (*n* = 1), Papua New Guinea (*n* = 1) and the United States (*n* = 1); ^3^ Maternal education level ≥Australian year 12 high school certificate; ^4^ IRSAD, index of relative socioeconomic advantage and disadvantage; ^5^ Relative disadvantage and lack of advantage based on postcode (IRSAD decile ≤ 5), ([25]); ^6^ Median; 25th and 75th percentiles in parentheses (all such values).

The BP and growth characteristics of children are summarized in Table 2. Compared with female children, male children became significantly heavier (*p* < 0.01) and longer (*p* < 0.05) between birth and 24 months, and had a larger BMI between 3 and 12 months (*p* < 0.01). No significant differences were found between child gender and BP measurements.

**Table 2:** Characteristics of children in the Women and Their Children’s Health Study.

Child Characteristics	Males	Females	*P* ^1^
**Birth**			
Gender [*n* (%)]	67 (51.9)	62 (48.1)	0.66
Gestational age (week)	39.5 ± 1.3 ^2^	39.3 ± 2.1	0.45
Birthweight (g)	3618.7 ± 552.7	3318.2 ± 591.2	0.003
Length (cm)	51.9 ± 2.8	50.3 ± 4.0	0.01
**3 months**			
*n*	64	60	
Weight (kg)	6.5 ± 0.8	5.7 ± 0.7	<0.001
Length (cm)	63.0 ± 2.5	60.6 ± 2.8	<0.001
BMI	16.1 ± 1.5	15.2 ± 1.5	<0.001
BMI *z*-score	−0.5 (−1.2, 0.2)	−0.7 (−1.7, −0.1)	0.26
*n*	20	21	
Systolic blood pressure (mmHg)	96.0 (82.5, 104.5) ^3^	96.0 (86.0, 103.0)	0.77
Diastolic blood pressure (mmHg)	60.0 (34.0, 66.5)	55.0 (47.0, 59.0)	0.68
**6 months**			
*n*	61	53	
Weight	8.2 ± 1.1	7.3 ± 1.0	<0.001
Length	69.3 ± 2.9	66.4 ± 3.0	<0.001
BMI	17.0 ± 1.4	16.5 ± 1.5	0.03
BMI *z*-score	−0.2 (−0.9, 0.4)	−0.3 (−0.9, 0.3)	0.72
*n*	36	36	
Systolic blood pressure (mmHg)	101.0 (96.0, 108.0)	103.0 (95.5, 109.0)	0.78
Diastolic blood pressure (mmHg)	57.0 (51.0, 65.5)	52.5 (45.5, 62.0)	0.30
**9 months**			
*n*	54	56	
Weight	9.5 ± 1.4	8.5 ± 1.0	<0.001
Length	73.9 ± 2.9	71.3 ± 2.9	<0.001
BMI	17.1 ± 1.7	16.6 ± 1.2	0.03
BMI *z*-score	−0.1 (−0.9, 0.8)	−0.04 (−0.7, 0.5)	0.76
*n*	40	48	
Systolic blood pressure (mmHg)	103.0 (98.0, 109.0)	101.5 (93.5, 110.0)	0.78
Diastolic blood pressure (mmHg)	55.5 (49.5, 62.0)	56.5 (46.0, 61.5)	0.54
**12 months**			
*n*	57	55	
Weight	10.5 ± 1.3	9.3 ± 1.0	<0.001
Length	78.0 ± 3.3	75.1 ± 2.9	<0.001
BMI	17.1 ± 1.4	16.4 ± 0.9	0.001
BMI *z*-score	0.1 (−0.5, 0.8)	0.1 (−0.5, 0.5)	0.24
*n*	37	38	
Systolic blood pressure (mmHg)	98.0 (94.0, 106.0)	98.5 (94.0, 108.0)	0.81
Diastolic blood pressure (mmHg)	58.0 (50.0, 66.0)	56.0 (46.0, 62.0)	0.59
**24 months**			
*n*	47	44	
Weight	13.5 ± 1.6	12.6 ± 1.8	0.009
Length	89.1 ± 4.9	86.7 ± 4.2	0.02
BMI	17.0 ± 1.3	16.7 ± 1.3	0.37
BMI *z*-score	1.0 (−0.1, 1.4)	0.7 (0.06, 1.2)	0.97
*n*	35	32	
Systolic blood pressure (mmHg)	100.0 (96.0, 105.5)	98.0 (92.0, 104.0)	0.13
Diastolic blood pressure (mmHg)	61.0 (56.0, 65.0)	61.0 (58.0, 64.5)	0.63
**36 months**			
*n*	42	38	
Weight	15.4 ± 1.8	14.4 ± 2.8	0.06
Length	97.4 ± 5.7	95.0 ± 7.3	0.11
BMI	16.1 ± 1.4	15.8 ± 1.5	0.34
BMI *z*-score	0.4 (−0.5, 1.3)	0.2 (−0.3, 0.7)	0.60
*n*	36	33	
Systolic blood pressure (mmHg)	103.0 (98.5, 111.0)	104.0 (97.0, 113.0)	0.87
Diastolic blood pressure (mmHg)	63.0 (59.5, 66.0)	62.0 (58.0, 67.0)	0.90
**48 months**			
*n*	40	45	
Weight	17.7 ± 2.1	17.4 ± 3.0	0.61
Length	105.3 ± 7.4	103.6 ± 5.8	0.24
BMI	15.1 ± 1.3	16.1 ± 2.5	0.61
BMI *z*-score	0.4 (−0.2, 1.3)	0.3 (−1.2, 0.8)	0.69
*n*	26	28	
Systolic blood pressure (mmHg)	101.0 (95.0, 114.0)	106.0 (94.0, 115.0)	0.72
Diastolic blood pressure (mmHg)	65.0 (59.0, 66.0)	65.0 (58.5, 67.5)	0.89
Systolic blood pressure up to 4 years (mmHg)	101.5 (97.0, 106.3)	101.6 (94.7, 106.3)	0.78
Diastolic blood pressure up to 4 years (mmHg)	60.3 (55.3, 63.5)	57.0 (50.3, 61.0)	0.01

^1^ *p* Values were derived by 2-sample *t* tests or the Kruskal-Wallis test; ^2^ Mean ± SD (all such values); ^3^ Median; 25th and 75th percentiles in parentheses (all such values).

Maternal dietary composition during pregnancy is summarized in Table 3. Results of the mixed-model regression analyses examining relationships between pregnancy diet and child systolic BP up to age 4 years are summarized in Table 4. The components of maternal diet associated with child systolic BP included: PUFA (% of energy), specifically *n*-6 fatty acids (% of energy), and the P:C ratio (Table 4). Median (25p, 75p) P:C ratio was 0.43 (0.38, 0.48). Therefore, for each 0.1 unit decrease in the P:C ratio during pregnancy, child systolic BP increased by 1.41 mmHg (Table 4). Similar findings were observed in the comparison of energy-adjusted values (Table 4). In subgroup analyses, maternal smoking status did not have a significant effect on childhood systolic BP (data not shown). There were no relationships between maternal pregnancy diet and child diastolic BP up to 4 years.

**Table 3:** Maternal dietary composition during pregnancy (*n* = 129) ^1^.

Absolute Values	Grams	% of Energy
Protein	81.4 (64.8, 105.6)	19.1 (17.4, 20.9)
Total fat	73.0 (57.7, 95.8)	37.6 (34.7, 40.0)
SFA	31.1 (23.2, 41.7)	15.9 (13.6, 18.4)
PUFA	11.2 (7.8, 13.7)	5.0 (4.0, 6.2)
*n*-3 Fatty acids	1.4 (1.0, 1.7)	0.7 (0.6, 0.8)
*n*-6 Fatty acids	9.2 (6.2, 11.9)	4.2 (3.3, 5.3)
MUFA	25.4 (19.5, 33.2)	13.0 (11.8, 13.9)
Total carbohydrate	185.3 (153.7, 244.0)	41.5 (39.3, 44.4)
Sugars	92.1 (72.2, 115.1)	19.5 (17.0, 22.1)
Starch	99.2 (79.3, 125.1)	21.2 (19.8, 23.5)
Fibre	19.2 (14.5, 24.9)	2.1 (1.7, 2.4)
Energy (kJ)	7298.4 (5890.1, 9234.2)	
P:C ratio	0.43 (0.39, 0.48)	
*n*-6:*n*-3 ratio	6.38 (5.22, 7.78)	
Energy-adjusted values	Grams	
Protein	121.9 (89.3, 128.6)	
Total fat	103.7 (97.4, 109.1)	
SFA	44.3 (39.6, 49.2)	
PUFA	13.5 (11.5, 16.2)	
*n*-3 Fatty acids	1.9 (1.7, 2.1)	
*n*-6 Fatty acids	11.1 (9.6, 13.9)	
MUFA	36.3 (34.4, 38.2)	
Total carbohydrate	250.0 (240.0, 264.3)	
Sugars	117.9 (106.1, 129.7)	
Starch	128.2 (116.4, 137.4)	
Fibre	26.7 (23.4, 30.0)	

P:C, protein-to-carbohydrate. *n*-6:*n*-3, omega-6-to-omega-3. ^1^ All values are medians; 25th and 75th percentiles in parentheses.

**Table 4:** Mixed-model regression analysis of the associations between diet during pregnancy and child systolic blood pressure up to age 4 years. Results presented for all dietary data (*n* = 129) ^1^.

	Systolic Blood Pressure (mmHg)								
	Crude model			Adjusted model (a)			Adjusted model (b)		
Maternal diet	β Coefficient	95% CI	*P* ^2^	β Coefficient	95% CI	*P* ^2^	β Coefficient	95% CI	*P* ^2^
Protein (% E)	−0.36	−0.88, 0.16	0.17	−0.39	−0.90, 0.12	0.13	−0.48	−0.99, 0.03	0.07
Polyunsaturated fat (% E)	0.69	−0.02, 1.41	0.06	0.68	−0.04, 1.40	0.06	0.73	0.003, 1.45	0.05
Omega-6 fatty acids (% E)	**0.84**	**0.05, 1.64**	**0.04**	**0.86**	**0.06, 1.66**	**0.04**	**0.89**	**0.09, 1.69**	**0.03**
Fibre (g)	0.09	−0.06, 0.24	0.24	0.26	0.001, 0.53	0.05	0.27	0.002, 0.54	0.05
P:C ratio	−10.93	−24.53, 2.67	0.12	−12.23	−25.70, 1.24	0.07	**−14.14**	**−27.68, −0.60**	**0.04**
*Energy adjusted values*									
Protein (g)	−0.07	−0.16, 0.02	0.13	−0.08	−0.16, 0.01	0.09	−0.09	−0.17, 0.003	0.06
Polyunsaturated fat (g)	**0.43**	**0.06, 0.80**	**0.02**	**0.42**	**0.05, 0.80**	**0.03**	**0.44**	**0.06, 0.81**	**0.02**
Omega-6 fatty acids (g)	**0.50**	**0.10, 0.90**	**0.02**	**0.50**	**0.09, 0.90**	**0.02**	**0.51**	**0.10, 0.92**	**0.01**
Fibre (g)	0.20	−0.07, 0.46	0.14	0.27	0.01, 0.53	0.05	0.27	0.01, 0.54	0.05

P:C, protein to carbohydrate. % E, percentage of energy. ^1^ Analysis models: (a) adjusted for maternal energy intake and child birthweight; (b) adjusted for maternal energy intake, child birthweight and child BMI *z*-score. Energy-yielding dietary variables not presented in the table above did not return any significant results for any model. ^2^ *P*-values were derived by linear mixed-model regression analyses.

**Figure 1:**
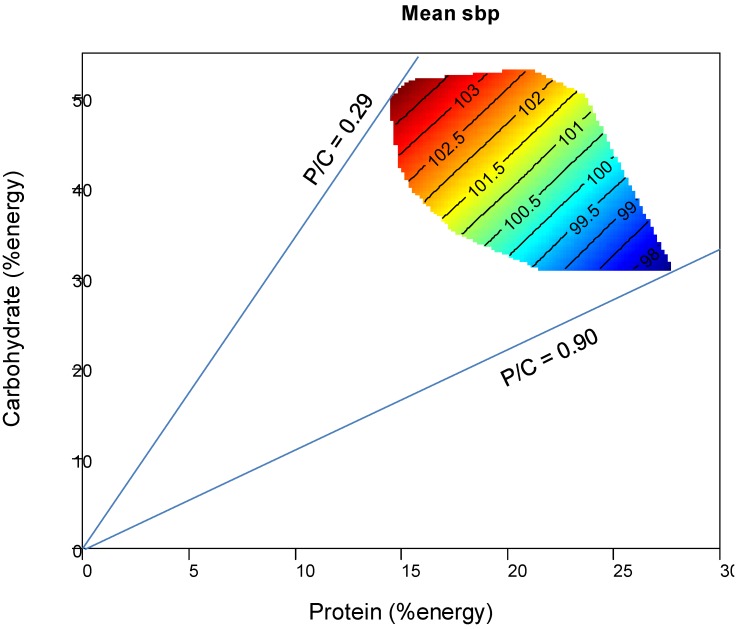
Effects of maternal macronutrient intake during pregnancy on child mean systolic blood pressure up to four years. Plotted onto arrays of maternal dietary macronutrient composition points are fitted surfaces for the response variable (child mean systolic blood pressure). The isolines for the child mean systolic blood pressure rise in elevation from dark blue to dark red. A maternal diet with a P:C ratio of 0.29 corresponded with a child systolic BP of 104.5, compared with a systolic BP of 97.5 for a P:C ratio of 0.9. Child systolic BP was greatest at low proportions of dietary protein (<16% of energy) and high carbohydrate (>40% of energy) intakes.

The association between maternal P:C ratio during pregnancy and child mean systolic BP up to 4 years is presented in Figure 1. The surface plot highlighted that mean child systolic BP remained constant with changing proportions of dietary fat, but was influenced by the P:C ratio of maternal diet during pregnancy. A maternal diet with a P:C ratio of 0.29 corresponded with a child systolic BP of 104.5, compared with a systolic BP of 97.5 for a P:C ratio of 0.9. Child systolic BP was greatest at low proportions of dietary protein (<16% of energy) and high carbohydrate (>40% of energy) intakes.

## 4. Discussion

This is the first prospective study to longitudinally examine the relationship between maternal diet during pregnancy and the trajectories of child BP up to four years. Results indicate that lower P:C ratios in maternal diet are associated with increased child systolic BP up to four years of age. Childhood systolic BP was also positively associated with maternal intake of PUFA and *n*-6 fatty acids, despite remaining constant with changing total fat intake.

Strong evidence supports the tracking of BP from childhood to adult life [4]. Women who gave birth during the Dutch famine in 1944–1945 illustrate that offspring BP as young adults was inversely associated with the P:C ratio of the average ration during the third trimester, but not associated with any absolute measure of intake [26]. This suggests that long-term health may be linked more strongly to the macronutrient ratio of maternal diet as opposed to absolute intakes. This study is the first to confirm similar findings in children. Mean childhood BP measures were within normal ranges (*i.e.*, below the 90th percentile) [27]. However, mean systolic BP and diastolic BP measures within this study were significantly higher than those reported by other contemporary birth cohorts [11,13]. The most likely explanation for these higher values is that BP was only measured on one occasion at each time point rather than an average of the two or three values used in other studies. The P:C ratio may further influence non-energy providing micronutrients that may affect childhood BP. Intervention studies have shown that a modest increase in dietary protein (5.3% of energy) with a corresponding reduction in carbohydrate subsequently decreased sodium intake (25mmol/day) in adults, in conjunction with a reduction in blood pressure [28]. Macronutrient ratios may influence offspring health and disease [16]. Future research is required to include the P:C ratio when evaluating the influence of maternal macronutrient composition during pregnancy on offspring health outcomes, and consider its impact on micronutrient intakes.

While potential molecular mechanisms related to our findings are poorly understood, animal models provide important mechanistic insights and suggest that nutritional deprivation or excess during pregnancy may stimulate epigenetic processes [5,13]. These events lead to both acute changes in offspring tissue structure and organ development, and permanent alterations in gene expression through DNA and histone methylation or histone acetylation [29,30], with subsequent cardio-metabolic consequences [5]. Rodent and sheep models support findings in the current study and provide strong evidence of a relationship between maternal protein intake during pregnancy and offspring cardiovascular function [6,8,31]. Offspring of nutritionally deprived animals commonly display elevated BP, while offspring exposed to protein or energy restriction during pregnancy show abnormal vascular function in isolated resistance arteries [32]. Additional consequences include increased sympathetic outflow [6], possibly due to altered interactions between the aberrant central signaling pathways in the renin-angiotensin system and sympathetic efferent activity [32]. While others have suggested mechanistic changes in renal sodium transport, including altered mRNA expression of α_1_- and β_1_- Na^+^/K^+^ ATPase and renal tubular bumetanide-sensitive and thiazidesensitive sodium co-transporters [5]. However, evidence for associations between maternal diet during pregnancy and offspring cardiovascular function in human cohorts, while valuable, is indirect. Causality cannot be inferred from this study because of its observational nature and the possibility of residual confounding cannot be excluded. Therefore, the effect of variations in maternal macronutrient composition requires further investigation using (i) animal model interventions; (ii) observational studies using statistical approaches such as propensity score analysis, to address residual confounding; or (iii) opportunistic examination within interventions targeting maternal nutrition to improve pregnancy dietary intake.

Compelling arguments have emerged from animal models of dietary balance (specifically the ratio of dietary P:C) regarding nutritional targets for optimal health and ageing [16]. The balance of macronutrients, rather than the absolute intake of a single nutrient, is a key determinant of lifespan [16]. In insect and mouse models, diets with decreased P:C were associated with increased lifespan and improved cardio-metabolic outcomes in later life [16]. These data are supported indirectly by a systematic review showing increased mortality in human studies associated with low carbohydrate diets [16], and a recent study showing increased mortality and cancer in humans on high protein diets [16]. Both high and low P:C diets have benefits and risks [33], which highlights an important role for macronutrient balance in optimizing the relationship between diet and disease risk. Recent evidence indicates that the interaction of a dominant protein appetite with weaker carbohydrate and fat appetites is a primary driver of total energy intake in mice and humans (termed “protein leverage”) [15]. The findings suggest that pregnant women may be driven to achieve a “target” percentage protein intake, as the median percentage of energy from protein throughout pregnancy within our sample was stable at 19% [14]. This potentially dominant appetite for protein during pregnancy may significantly influence the development of offspring BP in early childhood and thus play an important role in the development of pediatric and adult hypertension. Coupled with a severe decrease in P:C ratio being related to a greater tendency to store excess body fat and increased risk of decreased longevity associated with obesity [15], maternal macronutrient ratio may further increase the risk of elevated blood pressure through its influence on offspring adiposity [14]. Research investigating protein leverage during pregnancy, including the role of macronutrient balance (maternal prenatal and offspring postnatal diet) in the development of offspring blood pressure up until adulthood, and secondary outcomes such as increased adiposity, are required before pregnancy dietary recommendation to optimize childhood blood pressure can be formulated.

The role of maternal pregnancy PUFA intakes with development and regulation of child BP remains unclear. It is known that dietary PUFA intakes play a role in BP regulation and have usually been associated with beneficial effects [34]. However, emerging evidence suggests that PUFA classes (*n*-3 and *n*-6) have differential health effects, with *n*-6 PUFAs and PUFA ratios generating scientific debate regarding their role in human physiological processes [34]. Both classes of fatty acids have been shown to reduce BP in adult rats and hypertensive subjects [35,36]. However, significant reductions in BP in normotensive subjects have not been shown, nor have beneficial effects of PUFA on CVD risk markers been reported universally [36]. Arguments for reduced *n*-6 intakes surround the notion that inflammation plays a key role in CVD mechanisms [36]. Recent evidence from the Sydney Diet Heart Study reported that in men aged 30 to 59 years, substituting dietary *n*-6 linoleic acid (LA) in place of SFA showed no cardiovascular benefit and actually increased the risk of death from all causes, coronary heart disease and CVD [37]. The proposed mechanistic model linking *n*-6 PUFAs to cardiovascular pathogenesis is based on a diet-induced increase in the production of bioactive oxidized LA metabolites [37]. Oxidized LA metabolites are the most abundant oxidized fatty acids in oxidized low-density lipoprotein molecules. Ramsden and colleagues hypothesize that oxidative stress combined with diets high in *n*-6 LA facilitate this oxidation, leading to oxidized LA metabolite mediated atherosclerotic progression and increased cardiovascular mortality [37]. Increased circulating oxidized low-density lipoprotein has been reported in hypertensive men [38] and women with pre-eclampsia [39]. Limited research has been conducted in pregnancy. There is a lack of population-based research examining the relationship between maternal pregnancy PUFA intake and offspring BP. Results in the current study support a positive relationship between trajectory of childhood systolic BP up to age four years and maternal PUFA and *n*-6 fatty acids intakes during pregnancy, despite BP remaining constant with changing maternal total fat intake and being altered with P:C ratio. The influence of maternal diet on child BP may also be secondary to its influence on maternal BP during pregnancy. A higher maternal BP during pregnancy could reflect the mother’s own fetal experience, which consequently influences the intrauterine environment she provides for her children. Further research to elucidate the role of *n*-6 fatty acids in cardiovascular disease mechanisms, particularly maternal and child blood pressure, is warranted.

Limitations of the current study include the use of self-reported FFQ data to measure dietary intake. Dietary data is strengthened by the similarities between the daily mean energy intake reported in our study (8070 kJ/day) and that reported in a representative sample of pregnant women in the Australian Longitudinal Study on Women’s Health (7795 kJ/day) in 2003 [40]. Macronutrient distributions were also similar to the population nation data in pregnancy [40]. Physical activity data for children were unable to be obtained for our sample. BP measurements should be interpreted with caution. Automated BP monitors overestimate systolic BP and underestimate diastolic BP [41], and thus are not recommended for use in children in clinical settings where the accuracy of the absolute measurement is required [41]. In epidemiological studies where differences in BP between groups are more important than absolute levels, automated BP monitors such as the Dinamap are appropriate to reduce observer bias and digit preference [41]. Lastly, the WATCH study contained a higher proportion of women with post-school qualifications, socio-economic advantage and in a married or de facto relationship, but had a similar proportion of overweight/obesity and indigenous ethnicity compared to the Australian population [14]. Therefore, care should be taken in extrapolating results at the population level.

## 5. Conclusions

This study provides some evidence for an optimal maternal macronutrient profile associated with a healthy child BP. The development of systolic BP up to four years was positively associated with higher maternal PUFA intakes and a lower P:C ratio. Future focus on the maternal protein-to-carbohydrate ratio during pregnancy will be an important research area and may offer strategy to optimize offspring cardiovascular health. Further research has the potential to elucidate the role of maternal diet in childhood BP and in refining dietary recommendations provided to pregnant women in order to optimize offspring health long-term.
